# Predicting multi-class responses to preoperative chemoradiotherapy in rectal cancer patients

**DOI:** 10.1186/s13014-016-0623-9

**Published:** 2016-03-22

**Authors:** Jungsoo Gim, Yong Beom Cho, Hye Kyung Hong, Hee Cheol Kim, Seong Hyeon Yun, Hong-Gyun Wu, Seung-Yong Jeong, Je-Gun Joung, Taesung Park, Woong-Yang Park, Woo Yong Lee

**Affiliations:** Institute of Health and Environment, Seoul National University, Seoul, 151-742 Korea; Department of Statistics, College of Natural Science, Seoul National University, Seoul, 151-742 Korea; Department of Radiation Oncology, College of Medicine, Seoul National University, Seoul, 110-799 Korea; Department of Surgery, College of Medicine, Seoul National University, Seoul, 110-799 Korea; Department of Surgery, Samsung Medical Center, Sungkyunkwan University School of Medicine, Seoul, 135-710 Korea; Department of Molecular Cell Biology, Sungkyunkwan University School of Medicine, Seoul, 135-710 Korea; Samsung Medical Center, Samsung Genome Institute, Seoul, 135-710 Korea

**Keywords:** Prediction model, Rectal cancer, Chemoradiotherapy, Dworak classification, Microarray

## Abstract

**Background:**

Preoperative chemoradiotherapy (CRT) has become a widely used treatment for improving local control of disease and increasing survival rates of rectal cancer patients. We aimed to identify a set of genes that can be used to predict responses to CRT in patients with rectal cancer.

**Methods:**

Gene expression profiles of pre-therapeutic biopsy specimens obtained from 77 rectal cancer patients were analyzed using DNA microarrays. The response to CRT was determined using the Dworak tumor regression grade: grade 1 (minimal, MI), grade 2 (moderate, MO), grade 3 (near total, NT), or grade 4 (total, TO).

**Results:**

Top ranked genes for three different feature scores such as a *p*-value (pval), a rank product (rank), and a normalized product (norm) were selected to distinguish pre-defined groups such as complete responders (TO) from the MI, MO, and NT groups. Among five different classification algorithms, supporting vector machine (SVM) with the top 65 norm features performed at the highest accuracy for predicting MI using a 5-fold cross validation strategy. On the other hand, 98 pval features were selected for predicting TO by elastic net (EN). Finally we combined TO- and MI-finder models to build a three-class classification model and validated it using an independent dataset of rectal cancer mRNA expression.

**Conclusions:**

We identified MI- and TO-finders for predicting preoperative CRT responses, and validated these data using an independent public dataset. This stepwise prediction model requires further evaluation in clinical studies in order to develop personalized preoperative CRT in patients with rectal cancer.

**Electronic supplementary material:**

The online version of this article (doi:10.1186/s13014-016-0623-9) contains supplementary material, which is available to authorized users.

## Background

Treatment strategies for patients with rectal cancer have changed substantially in recent decades. Historically, postoperative chemoradiotherapy (CRT) was considered to be first-line therapy for stage II and III rectal cancers. However, preoperative CRT is now considered to be optimal therapy for locally advanced rectal cancer due to improved local control, reduced toxicity, and increased rates of sphincter preservation [1, 2].

Preoperative chemoradiotherapy (CRT) has been widely used as the treatment of choice for locally advanced rectal carcinomas [3, 4]. Radiotherapy works by inhibiting cell proliferation and inducing apoptosis in vitro, and inhibiting tumor growth in vivo [5, 6]. However, responses to radiotherapy differ among individuals with similar histologic backgrounds, and the essential determinants of these responses have yet to be studied. Thus, identifying the key factors that predict responses to radiotherapy before treatment could be helpful in that those patients predicted to have poor responses can undergo the initial surgery without preoperative CRT.

To date, several studies have investigated the response of rectal cancer to radiotherapy using gene expression microarrays [7–10]. In one report, pre-therapeutic biopsies from 30 patients with locally advanced rectal carcinomas were profiled. By grouping the radiation responses of patients defined as T-level downsizing, a set of 54 genes was found to be differentially expressed between responders and non-responders. Diagonal linear discriminant analysis (LDA) of the 54 genes was applied to predict responses to CRT. Using a leave-one-out cross validation (LOOCV) strategy, responses for 83 % of patients were correctly predicted.

Watanabe et al. [8] identified 33 genes that were differentially expressed in responders and non-responders as determined by histopathologic regression grading of surgically resected specimens from 52 rectal cancer patients. The prediction accuracy in this study was 88.6 % for the 17 test samples using the *k*-nearest-neighbor algorithm. Similarly, Kim et al. [7] and Rimkus et al. [10] reported 87 and 78.5 % prediction accuracies for 46 and 43 patients, respectively.

It is important to note, however, that there is very little overlap in the genes included in these studies, even when high prediction accuracies were reported [11]. This inconsistency may be due to use of different criteria in defining a response, small sample size, or different origins of responses. Additionally, all of the previous studies formulated models that predicted a two-class classification system by applying a specific algorithm with a simple feature selection procedure using a *p*-value threshold. Taken together, these issues indicate that no optimal system for gene identification has yet been developed for incorporation into clinical practice.

Our objective was to build a clinically feasible model that predicts multi-class responses to CRT. A schematic representation of whole analysis flow is shown in Table 1. We classified responses of patients according to Dworak grade using 77 patient samples, which is the largest sample size reported in the published literature. We then defined three different feature scores to identify a novel set of genes for predicting responses. Various classification algorithms were applied to this set of genes to build a complete regression (TO) prediction model and a minimal regression (MI) prediction model. From the most accurate models, we established a novel strategy for predicting multiple responses to CRT by applying TO and MI models sequentially. To date, this is the first trial to predict multi-class responses to CRT. Finally, we validated our model using a published dataset from an independent source.

**Table 1:** Schematic representation of the overall analysis flow

1. Collecting 77 rectal cancer samples with clinical features
2. Gene expression profiling with Affymetrix ST1.0 array
3. Feature selection by pval, norm and rank
4. Designing most accurate prediction models for MI or TO
5. Testing the prediction models for MI or TO
6. Multi-class prediction model
7. Internal validation of prediction model
8. External validation of the prediction model

## Methods

### Patient samples and response classification

This study was approved by the Institutional Review Boards (IRBs) at Samsung Medical Center (IRB No. SMC 2009-10-067). Written informed consent for participation in this study was obtained from the patient. A total of 77 rectal cancer patients who underwent preoperative CRT were included in this study. Various clinical information such as UICC stage and Dworak grade are summarized in Table 2 and more detailed information is available in supplementary Additional file 1: Table S1. Responses to CRT were determined according to Dworak tumor regression grade. Tumor regression grades were classified into five groups: grade 0 (no regression), grade 1 (minimal regression, MI), grade 2 (moderate regression, MO), grade 3 (near total regression, NT), and grade 4 (total regression, TO). A total of 10, 36, 13, and 18 patients were classified as MI, MO, NT, and TO, respectively. Tumor stage was determined according to the guidelines set forth by the International Union Against Cancer (UICC).

**Table 2:** Clinicopathologic features and responses to preoperative CRT

Parameters	Value
Number of patients	77
Median age (yrs, range)	56, 33–76
Sex	
Male	54
Female	23
Histological subtype	
Adenocarcinoma	72
Mucinous	3
Signet ring cell	2
Median interval to surgery (days, range)	56, 41–76
CEA (ng/ml)	
Before CRT	4.7 ± 6.6
After CRT	1.9 ± 1.5
UICC stage after surgery	
0	16 (20.8 %)
I	18 (23.4 %)
II	18 (23.4 %)
III	22 (28.6 %)
IV	3 (3.9 %)
Number of lymph nodes	11.8 ± 6.6
Lymphatic invasion (+)	17 (22.1 %)
Vascular invasion (+)	5 (6.5 %)
Perineural invasion (+)	4 (5.2 %)
Dworak regression grade	
Grade 1	10 (13.0 %)
Grade 2	36 (46.8 %)
Grade 3	13 (16.9 %)
Grade 4	18 (23.4 %)

### RNA isolation and microarray procedures

Total RNA was extracted from tumor tissue using TRIzol reagent (Invitrogen, Carlsbad, CA), and the collected RNA was purified using RNeasy mini kits (Qiagen, Valencia, CA). The purity and concentration of RNA were determined using a Bioanalyzer (Agilent Technologies, Santa Clara, CA). The RNA was amplified and labelled according to the Affymetrix GeneChip Whole Transcript (WT) Sense Target Labelling protocol. The resultant labeled cDNA was hybridized to Affymetrix Human Gene 1.0 ST arrays and scanned. The R program from CEL file preprocessing was used for all statistical analyses. The expression data obtained from each microarray was normalized using a Robust Multislide Array (RMA) normalization algorithm. Raw and expression microarray datasets are available upon request.

### Statistical analysis

#### Feature scores

We first placed the patients into groups labeled TO or other, and MI or other. We then used a two-sample Welch’s *t*-test with unequal variances to determine which genes were differentially expressed between groups. The feature score (FS) was defined as shown below. For genes *i*, *p*_*i*_, and *d*_*i*_ the *p*-value and effect size were obtained from predefined group comparisons. *P*-value based (‘pval’), rank product based (‘rank’), and normalized value product based (‘norm’) feature scores were calculated.

$$ FS=\left\{\begin{array}{c}\hfill -{ \log}_{10}\left({p}_i\right),\hfill \\ {}\hfill rank\left(-{ \log}_{10}\left({p}_i\right)\right)\times rank\left({d}_i\right),\hfill \\ {}\hfill norm\left(-{ \log}_{10}\left({p}_i\right)\right)\times norm\left({d}_i\right),\hfill \end{array}\right.\ \begin{array}{c}\hfill \mathrm{pval}\hfill \\ {}\hfill \mathrm{rank}\hfill \\ {}\hfill \mathrm{norm}\hfill \end{array} $$

where, $$ norm\;\left({x}_i\right)=\frac{x_i- \min\;(x)}{ \max\;(x)- \min\;(x)}\;. $$

#### Classification models

To build prediction models, we applied multiple classification algorithms using varying numbers of features based on ‘pval’, ‘rank’, and ‘norm’ scores. Samples from 77 patients were divided into a training set and a test set. We applied 5 different algorithms including support vector machine (SVM), random forest (RF), elastic-net (EN) [12], linear discriminant analysis (LDA) and *k-*nearest neighbor (*k*NN) [13] with *k* = 1, 3, and 5. The TO and the MI predictors were built by applying all algorithms using varying numbers of features with different feature scores to two different classification problems (i.e., TO vs. other and MI vs. other).

#### Model selection

We consider *iid* data (*x*_1_ , *y*_1_),⋯, (*x*_*n*_ , *y*_*n*_) where *x*_*i*_ = (*x*_*i*1_,⋯, *x*_*id*_) ∈ *X* ⊂ ℝ^*d*^ is a *d*-dimensional expression vector and ${Y}_1$ denotes values of a specific class in some finite set *Y*. A classification rule is a function: *h* : *X* → *Y*. Our goal was to choose the optimal classification rule or classifier *ĥ* to minimize the training error. We adopted a 5-fold cross-validation (CV) scheme to estimate the prediction accuracy for each classification method and select the most accurate model. Note that the CV scheme used in this work is not a conventional CV approach, in which only training data is used for selecting features and training a classifier. Since the number of sample was not large enough, we evaluated the feature scores with whole dataset to reliably select the important features. This might cause biased estimate of the true error of the model prediction. Thus we applied the final model to an independent publicly available dataset.

#### Sequential multiclass prediction

Multiclass (minimal, moderate, or total response) prediction was conducted by applying the best MI and TO prediction models sequentially. With SVMs, reducing the single multiclass problem into several binary classification problems is likely a better approach [14]. We first applied the TO model to an individual to predict whether the individual was classified into that group. If not, the MI model was applied and the final conclusion was made according to the MI prediction result.

#### Application to independent published dataset

An independent test set was obtained from the previous study which classified the patient responses using Dworak criteria [7]. Thus, we can apply our model without a response classification criteria compatibility issue. Because of the platform difference, however, direct application of our model to data reported in [7] had two limitations: the limited number of overlapping features and the different expression scale. To resolve these issues, we first matched the features (genes) using “HGNC gene symbols” and subsequently applied quantile normalization of these genes using average values of the same ranked genes in our dataset. We identified 38 (of 65) and 32 (of 98) common genes for the MI and TO models. Since only 70 of 163 of the best model features were available, the best model found using 77 samples could not be directly applied to the dataset reported in [7]. Therefore, we rebuilt the model with common genes to better predict responses to CRT.

## Results

### Three feature score types and their characteristics

To identify molecular features of CRT responses, we analysed whole gene expression profiles of 77 rectal cancer biopsy specimens. The overall scheme for a multi-class prediction model is summarized in Table 1. We divided patients into TO versus other, or MI versus other. For each comparison, we obtained *p*-values and effect sizes for all genes. From these two values, we defined three different feature scores including *p*-value, normalized product, and rank product.

**Fig. 1:**
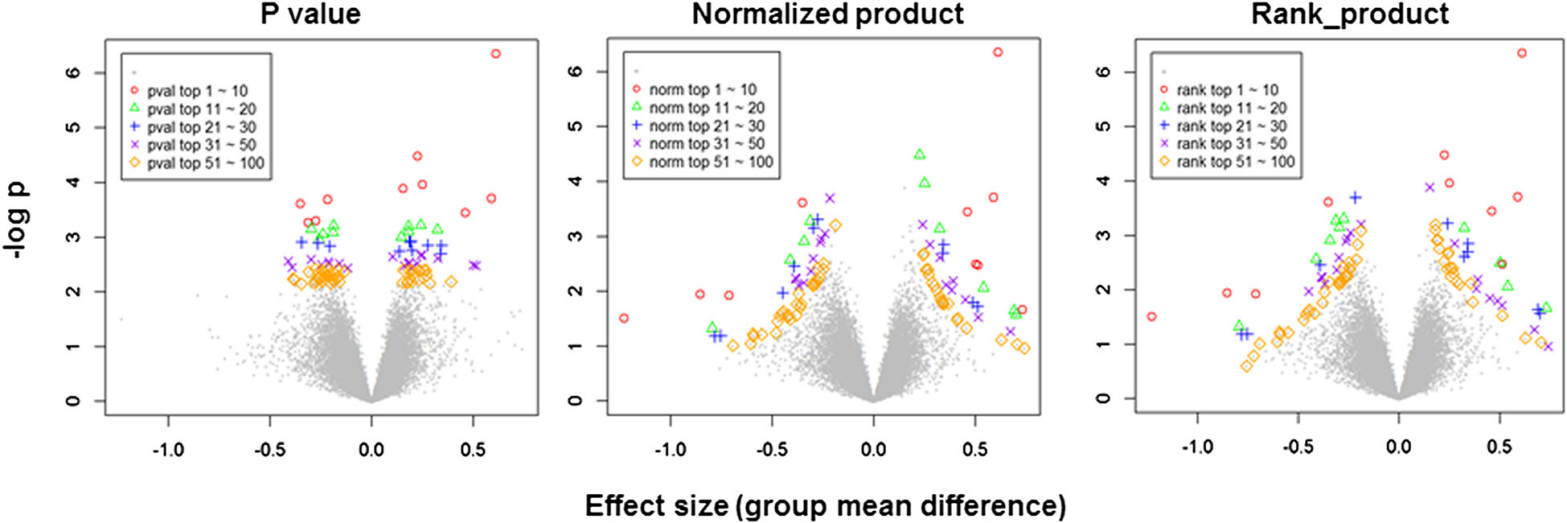
Characteristics of features selected by three different scores. All genes are represented in volcano plot. Gene with top 100 highest feature score are depicted with different color and shape while others with grey color. *P*-value based feature score (‘pval’, left), normalized product based (‘norm’, middle) and rank product based feature scores (‘rank’, right) are shown

Characteristics of the features with different FS criteria used for TO prediction are shown in Fig. 1. *P*-value based (‘pval’) features were selected using the lowest ‘pval’ criterion. Unlike the ‘pval’ score, the normalized product (‘norm’) and rank product (‘rank’) scores with higher *p*-values (less significant) were selected if they also had larger effect sizes. Thus, in selecting features with higher ‘norm’ and ‘rank’ scores, both the effect size and the *p*-value were considered. A similar pattern was observed for features predictive of MI.

To investigate the effect of prediction performance of three FS criteria, we evaluated average accuracy across the models with different number of features for each classification algorithm (Table 3). Among these three FS measures, ‘pval’ showed the superior to other measures. For MI prediction, 5 out of 7 different methods with ‘norm’ FS type were found to be the best performer. For TO prediction, however, all the highest values were observed with ‘pval’ FS. In addition, SVM with ‘pval’ FS showed the most stable and the highest average accuracy for both MI and TO prediction (Table 3).

**Table 3:** Average accuracy across the different number of features

Predictors	FS	Algorithm						
		SVM	RF	EN	LDA	kNN1	kNN3	kNN5
MI	pval	0.96	0.89	0.89	0.77	0.88	0.87	0.87
	norm	0.96	0.88	0.90	0.82	0.85	0.89	0.90
	rank	0.95	0.88	0.89	0.81	0.85	0.88	0.90
TO	pval	0.87	0.83	0.87	0.72	0.77	0.78	0.78
	norm	0.81	0.80	0.74	0.67	0.72	0.73	0.74
	rank	0.81	0.81	0.75	0.67	0.72	0.74	0.75

### TO and MI prediction models

To find the most accurate TO and MI prediction models, we conducted various classification analyses using multiple features. Rather than using the leave-one out cross validation (LOOCV) that many similar studies have used, we adopted a 5-fold cross-validation strategy to prevent overfitting (i.e., making the model less fit to the sample data but a better fit with new data). Classification accuracy (1 ‐ error) was evaluated using five different classification algorithms: support vector machine (SVM), random forest (RF), elastic net (EN), linear discriminant analysis (LDA), and k nearest neighbor (kNN, with k=1, 3, and 5).

**Fig. 2:**
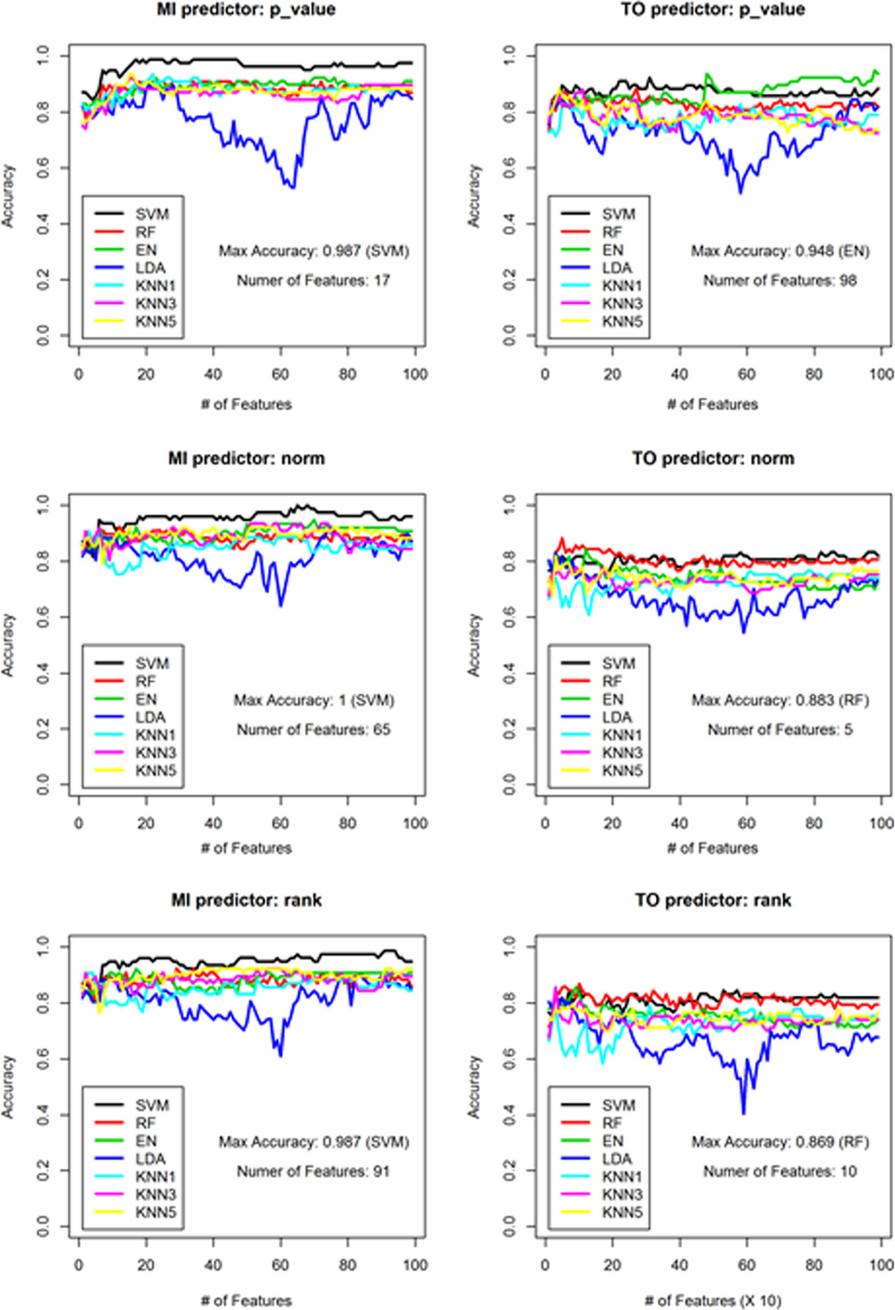
Binary-class prediction accuracy for MI and TO. Column and row of whole figure represent prediction class (MI or TO) and feature score used (pval, norm, or rank), respectively. For each panel, different color denotes different classification algorithm. Maximum accuracy among the algorithms and the number of feature used for the maximum value are also depicted in each panel

As can be seen in Fig. 2, in most cases, prediction performances were consistently around 85 % regardless of the classification algorithm, feature score type, or number of features. LDA, however, had poor prediction performance with an accuracy of approximately 50 % when more than 30 features were used (Fig. 2). Since some of genes may be highly correlated, collinearity among an increasing number of features may impede the performance of the LDA algorithm.

The best accuracy rates for MI and TO predictions were attained using SVM and EN, respectively. Using the SVM classification model, the prediction accuracy for MI was 100 % with 65 ‘norm’ feature score genes (Fig. 2). For TO predictions, the EN algorithm achieved a 94.8 % accuracy rate with 98 ‘pval’ feature score genes (Fig. 2). Note that SVM showed the most stably high prediction rate performances regardless of the number of features used.

### Gene function annotation of the best TO and MI predictors

To investigate the biological functions of the genes that are predictive of TO and MI, we conducted gene-set enrichment analysis using DAVID [15]. With the gene ontology as a gene set, we observed that the genes predictive of TO were enriched in the regulation of cell migration, cell maturation, and cell death, whereas the genes predictive of MI were involved in protein localization and protein transport (Table 4). With the pathway as a gene set, N-Glycan biosynthesis and Oocyte meiosis pathways were enriched by the genes predictive of TO and MI, respectively (Table 4). These two pathways are associated with responses to irradiation [16, 17]. N-linked glycosylation in particular enhances the effects of radiation therapy and is advantageous for inhibition of tumor growth [17]. The whole list of genes and the results of gene set analysis can be found in Additional file 2: Table S2.

**Table 4:** Gene-set analysis with TO and MI predictors using DAVID (https://david.ncifcrf.gov/)

Predictors	GO	Term	*P*-value	Genes
TO	GO:0030334	Regulation of cell migration	0.046	BCAR1, RRAS, BDKRB1, APC
	GO:0048469	Cell maturation	0.049	HES1, TFCP2L1, MTCH1
	GO:0043065	Positive regulation of apoptosis	0.052	IFNA2, OBSCN, GSDMA, MTCH1, RPS27A, APC
	HSA00510	N-Glycan biosynthesis	0.021	MGAT1, RFT1, GANAB
MI	GO:0008104	Protein localization	0.010	SFT2D2, BLZF1, KIFAP3, BCAP29, NECAP1, GOSR1, SEC62, NSF, SRP9
	GO:0015031	Protein transport	0.015	SFT2D2, BLZF1, BCAP29, NECAP1, GOSR1, SEC62, NSF, SRP9
	GO:0045184	Establishment of protein localization	0.016	SFT2D2, BLZF1, BCAP29, NECAP1, GOSR1, SEC62, NSF, SRP9
	HSA04114	Oocyte meiosis	0.051	CCNE2, RPS6KA6, ITPR2

### A sequential approach model for multi-class prediction and external validation

**Fig. 3:**
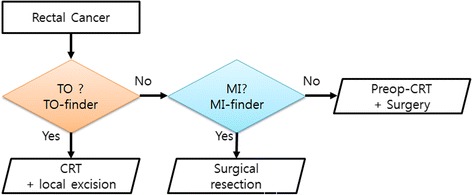
Sequential multi-class prediction. To predict the preoperative CRT response of a patient, TO predictor is performed and answers whether the patient is total response group or not. If yes, CRT is conducted, or MI predictor is performed and predicts whether the patient will show minimal response. According to the result of this step, clinician can decide a proper treatment of the patient

We next examined whether our two best models are practically useful in predicting multi-class responses to radiotherapy. To this end, we combined TO- and MI-finder models for a three-class prediction (Fig. 3). We used a sequential approach in applying the TO and MI models to both our dataset and validated the performance using a previously published dataset. Since not all of the genes used in our study were available in the dataset obtained in [7], we used overlapping genes included in both datasets. We found that the best TO prediction algorithm was EN. However, only a third of the previously described TO predictive genes were included in the dataset, and in cases in which there were fewer feature numbers, SVM was more accurate than EN (Fig. 2). Thus, our sequential approach model was designed to use the expression of 70 matched genes including 32 genes for TO prediction and 38 genes for MI prediction.

The sensitivities for predicting TO, MO (including NT), and MI using the sequential approach model among the 77 samples were 94.4, 100, and 90 %, respectively (Table 5). We defined sensitivity for each class as a correct prediction. To further test the validity of our model, we also applied it to an independent dataset [7]. The sensitivites for TO, MO and MI prediction were 86.6, 100, and 64.3 %, respectively (Table 5). We also confirmed that a sequential approach in the reverse order (MI prediction first, then TO prediction) did not change the prediction results in either dataset.

**Table 5:** Validation of multi-class prediction model

Internal validation		True		
		MI	MO	TO
PREDICTION	MI	9	0	0
	MO	1	49	1
	TO	0	0	17
External validation		True		
		MI	MO	TO
PREDICTION	MI	9	0	0
	MO	5	17	2
	TO	0	0	13

## Discussion

The objective of this study was to generate a clinically feasible model that predicts multi-class responses to CRT. In the past, one of the difficulties that impeded the development of such a model was the use of non-overlapping sets of genes across different studies, which may due to different definitions of response to CRT or the genetic diversity of rectal cancer [11, 18]. We sought to eliminate this issue by increasing our sample size and using a Dworak regression grade for distinct classification according to our three FS criteria. To date, this is the largest study that examines the genetic diversity of rectal cancer. The lack of independent prospective validation has also been an issue in previous studies, as many have used a LOOCV strategy to validate prediction accuracies due to small sample sizes (less than 50).

However, LOOCV may strengthen prediction accuracy by over-training with n ‐ 1 samples from the total sample pool. In our study, we applied a 5-fold cross-validation strategy to the 77 samples to achieve a higher degree of independence between training and test data. It is important for the clinician to classify the patient into the appropriate category, such as total response, intermediate response, or no response. To achieve this, we established the multi-class prediction model by adopting a novel sequential application of two separate prediction models. Using Dworak regression grade, the results of our multi-class prediction analysis resulted in highly accurate classification (97.4 %; Table 5). A relatively lower accuracy (84.67 %; Table 5) was obtained using the previously published independent dataset that included 46 patients. While these data are encouraging, prospective clinical trials using homogenous cohorts of patients (11) are needed to confirm the feasibility of using this model in clinical practice.

Some limitations are worth noting. Although our model was supported by independent dataset, conventionally cross-validation approach, in which features are selected on a training set, was not applied in our study due to a small sample size. Also we built our model based on univariate feature selection method without considering clinical predictors. Future work should therefore include follow-up work designed to evaluate whether our model and our approach are retained in more datasets and also whether the joint analysis of multiple genes, clinical variables, and interaction among them are necessary to be used to improve prediction performance.

## Conclusions

Historically postoperative chemoradiotherapy (CRT) was considered to be the first-line therapy for stage II and III rectal cancers. However, the preoperative CRT is now considered to be the optimal therapy regimen for locally advanced rectal cancer due to its improved local control, reduced toxicity, and increased rate of sphincter preservation. Our study established a clinically practical multi-class prediction model by adopting a novel strategy that applies two separate prediction models (MI and TO predictor) sequentially to a patient to predict the response. For promising clinical practice, we validated our model in a published dataset, which is completely independent dataset from ours. This study suggests a clinically plausible prediction model that correctly infers the preoperative CRT response of patients with high accuracy based on 163 gene signatures we identified. Larger prospective trials will be needed to confirm harder the validity of the present scheme.

## Additional files

Additional file 1: Table S1. Clinical information. (XLSX 19.5 kb) Additional file 2: Table S2. List of TO and MI genes used as the features for the prediction model. (XLSX 20.9 kb)

## References

[1] Randomised trial of surgery alone versus radiotherapy followed by surgery for potentially operable locally advanced rectal cancer. Medical Research Council Rectal Cancer Working Party. Lancet. 1996;348:1605–10. 8961989

[2] Park CH, Kim HC, Cho YB, Yun SH, Lee WY, Park YS (2011). Predicting tumor response after preoperative chemoradiation using clinical parameters in rectal cancer. World J Gastroenterol.

[3] Improved survival with preoperative radiotherapy in resectable rectal cancer. Swedish Rectal Cancer Trial. N Engl J Med. 1997;336:980–7. 10.1056/NEJM199704033361402 9091798

[4] Nagawa H, Muto T, Sunouchi K, Higuchi Y, Tsurita G, Watanabe T (2001). Randomized, controlled trial of lateral node dissection vs. Nerve-preserving resection in patients with rectal cancer after preoperative radiotherapy. Dis Colon Rectum.

[5] Ogawa K, Murayama S, Mori M (2007). Predicting the tumor response to radiotherapy using microarray analysis (review). Oncol Rep.

[6] Hendry JH, West CM (1997). Apoptosis and mitotic cell death: their relative contributions to normal-tissue and tumour radiation response. Int J Radiat Biol.

[7] Kim IJ, Lim SB, Kang HC, Chang HJ, Ahn SA, Park HW (2007). Microarray gene expression profiling for predicting complete response to preoperative chemoradiotherapy in patients with advanced rectal cancer. Dis Colon Rectum.

[8] Watanabe T, Komuro Y, Kiyomatsu T, Kanazawa T, Kazama Y, Tanaka J (2006). Prediction of sensitivity of rectal cancer cells in response to preoperative radiotherapy by DNA microarray analysis of gene expression profiles. Cancer Res.

[9] Ghadimi BM, Grade M, Difilippantonio MJ, Varma S, Simon R, Montagna C (2005). Effectiveness of gene expression profiling for response prediction of rectal adenocarcinomas to preoperative chemoradiotherapy. J Clin Oncol.

[10] Rimkus C, Friederichs J, Boulesteix AL, Theisen J, Mages J, Becker K (2008). Microarray-based prediction of tumor response to neoadjuvant radiochemotherapy of patients with locally advanced rectal cancer. Clin Gastroenterol Hepatol.

[11] Akiyoshi T, Kobunai T, Watanabe T (2012). Predicting the response to preoperative radiation or chemoradiation by a microarray analysis of the gene expression profiles in rectal cancer. Surg Today.

[12] Friedman J, Hastie T, Tibshirani R (2010). Regularization paths for generalized linear models via coordinate descent. J Stat Softw.

[13] Hsu CW, Lin CJ (2002). A comparison of methods for multiclass support vector machines. IEEE Trans Neural Netw.

[14] Chu W, Ong CJ, Keerthi SS (2005). An improved conjugate gradient scheme to the solution of least squares SVM. IEEE Trans Neural Netw.

[15] Huang da W, Sherman BT, Lempicki RA (2009). Systematic and integrative analysis of large gene lists using DAVID bioinformatics resources. Nat Protoc.

[16] Pesty A, Doussau M, Lahaye JB, Lefevre B (2010). Whole-body or isolated ovary (60)Co irradiation: effects on in vivo and in vitro folliculogenesis and oocyte maturation. Reprod Toxicol.

[17] Contessa JN, Bhojani MS, Freeze HH, Ross BD, Rehemtulla A, Lawrence TS (2010). Molecular imaging of N-linked glycosylation suggests glycan biosynthesis is a novel target for cancer therapy. Clin Cancer Res.

[18] Grade M, Wolff HA, Gaedcke J, Ghadimi BM (2012). The molecular basis of chemoradiosensitivity in rectal cancer: implications for personalized therapies. Langenbecks Arch Surg.

